# Cell derived nanovesicles for oral and craniofacial tissue regeneration

**DOI:** 10.1039/d6nr00456c

**Published:** 2026-04-23

**Authors:** Linna Zhong, Jeffrey S. Marschall, Kyungsup Shin, Hongli Sun

**Affiliations:** a Iowa Institute for Oral Health Research, University of Iowa College of Dentistry N409 DSB 801 Newton Road Iowa City IA 52242 USA hongli-sun@uiowa.edu +1 319-335-1217; b Department of Oral and Maxillofacial Surgery, University of Iowa College of Dentistry Iowa City IA 52242 USA; c Department of Orthodontics, University of Iowa College of Dentistry Iowa City IA 52242 USA

## Abstract

Oral and craniofacial diseases represent a significant global public health concern, profoundly impacting patient quality of life and imposing a substantial socioeconomic burden. While cell-based, particularly stem cell-based, regenerative strategies have shown promise in addressing the limitations of conventional therapies, they are also constrained by inherent challenges associated with cell therapy. Instead of relying on whole cells, cell-derived nanovesicles (CDNs), which inherit diverse biological functions from their parent cells, have emerged as a promising frontier in regenerative medicine. CDNs play a pivotal role in restoring microenvironmental homeostasis and modulating inflammation, thereby promoting angiogenesis and osteogenesis to support effective tissue regeneration. Furthermore, the therapeutic efficacy of CDNs can be enhanced through cell pretreatment and bioengineering strategies, such as cargo loading and surface modification. Owing to their ability to penetrate biological barriers, exhibit prolonged circulation, and achieve tissue-specific targeting, CDNs represent an advantageous drug delivery platform. Indeed, the development of engineered CDNs and hybrid composite systems has yielded excellent therapeutic outcomes by enhancing the precision and efficiency of drug delivery. This review systematically categorizes four major classes of CDNs, including exosomes (Exos), exosome mimetics (EMs), cell membrane nanovesicles (mNVs), and apoptotic extracellular vesicles (ApoEVs), evaluating their roles in treating craniofacial bone defects, osteoporosis, periodontitis, and dentin–pulp complex regeneration. Finally, we highlight the clinical potential of CDN-based therapies and outline future research directions for their application in oral and craniofacial tissue regeneration.

## Abstract 2

Oral and craniofacial diseases represent a significant global public health concern, profoundly impacting patient quality of life and imposing a substantial socioeconomic burden. Created in BioRender. Zhong, L. (2026) https://BioRender.com/v0qghgu.

## Graphical abstract

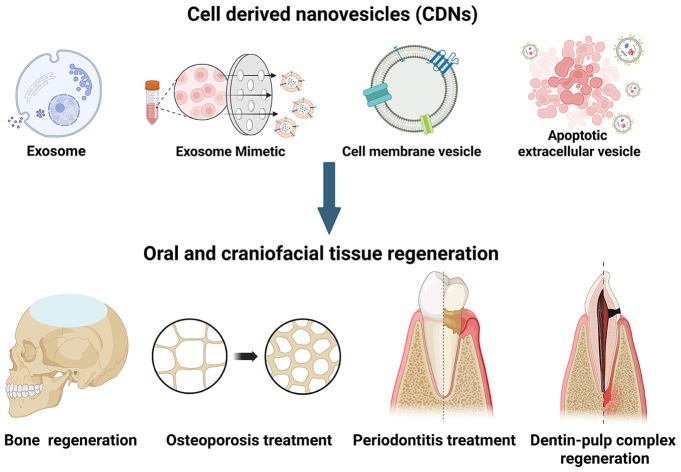

## 1. Introduction

Oral and craniofacial diseases pose a global threat to public health, presenting serious risks to human life expectancy and quality of life, which affect more than 3.5 billion people worldwide and result in US $387B due to direct costs globally in 2019.^1,2^ The craniofacial region is a complex structure composed of bone, muscle, salivary glands, teeth and periodontium, all of which are susceptible to a wide range of diseases and disorders.^3^ Craniofacial bone diseases and defects arising from trauma, congenital anomalies, and surgical resection significantly impair quality of life and impose a significant societal economic burden. Autologous bone grafting remains the gold standard for reconstructing large defects, but its clinical utility is limited by donor-site morbidity, restricted tissue availability, and significant postoperative bone resorption. Osteoporosis represents a growing medical and socioeconomic challenge, characterized by reduced bone mass and disrupted microarchitecture leading to fragility fractures, particularly in postmenopausal women and older adults.^4^ However, current therapeutic managements for osteoporosis have various side effects, among which medication-related osteonecrosis of the jaw (MRONJ) is a rare but most serious adverse effect of anti-resorptive agents (bisphosphonates and denosumab).^5^ Recent studies showed that bisphosphonates, one of the most prescribed anti-resorptive medications, can inhibit osteoclast bioactivities extremely, which is attributed to imbalanced bone remodeling and immunity dysfunction.^6^

Moreover, periodontal disease is a pathogen-induced chronic inflammatory disease characterized by progressive destruction of periodontal ligament and alveolar bone, leading to bone resorption, implant failure, and tooth loss.^7^ Severe periodontitis affects more than 700 million people globally, and demonstrates a close relationship to systemic diseases, such as diabetes mellitus, cardiovascular diseases and Alzheimer's disease, posing an urgent need for efficient periodontitis management.^8,9^ Current treatments for periodontal disease include scaling and root planning (SRP), local laser therapy, and surgical treatments, such as guided tissue regeneration (GTR). However, significant challenges remain in periodontal tissue regeneration after conventional therapies, largely due to the persistent inflammatory microenvironment and limited regenerative capacity to reverse alveolar bone loss.^10^ Additionally, pulpitis and pulp necrosis are common oral diseases, and the current treatment is root canal therapy, where the inflamed pulp is removed and filled with artificial material, resulting in a nonvital tooth with impaired functions.^11^

Conventional therapies for oral and craniofacial diseases often fail to achieve satisfactory outcomes and long-term functional success because they lack sufficient regenerative capacity. Therefore, there is a significant demand to develop advanced regenerative medicine strategies that surpass the limitations of traditional treatments. The key to these next-generation treatments for oral and craniofacial diseases is the ability to effectively restore the microenvironment homeostasis and modulate inflammation toward a pro-healing phenotype, thereby facilitating angiogenesis and osteogenesis for comprehensive soft and hard tissue regeneration. According to the principles of tissue engineering, which aim to mimic the natural tissue-healing process, the foundational pillars include scaffolds, cells, and growth factors, working together to guide cellular differentiation and support tissue repair.^12^ Previous research indicated that stem cells loaded scaffolds, usually mesenchymal stem/stromal cells (MSCs), showed great therapeutic potentials in the regenerative processes.^13^ Although MSCs were traditionally expected to differentiate into functional tissues, their survival rate after implantation is very low, and cell transplantation can also trigger immune reactions. Rather than acting through direct differentiation, recent findings indicate that the therapeutic benefits of MSCs are predominantly mediated by their paracrine signaling activity. Specifically, growing evidence highlighted the role of MSC-derived exosomes and apoptotic extracellular vesicles in regulating the immune response and recruiting local cells to regenerate damaged tissue.^14,15^

**Fig. 1:**
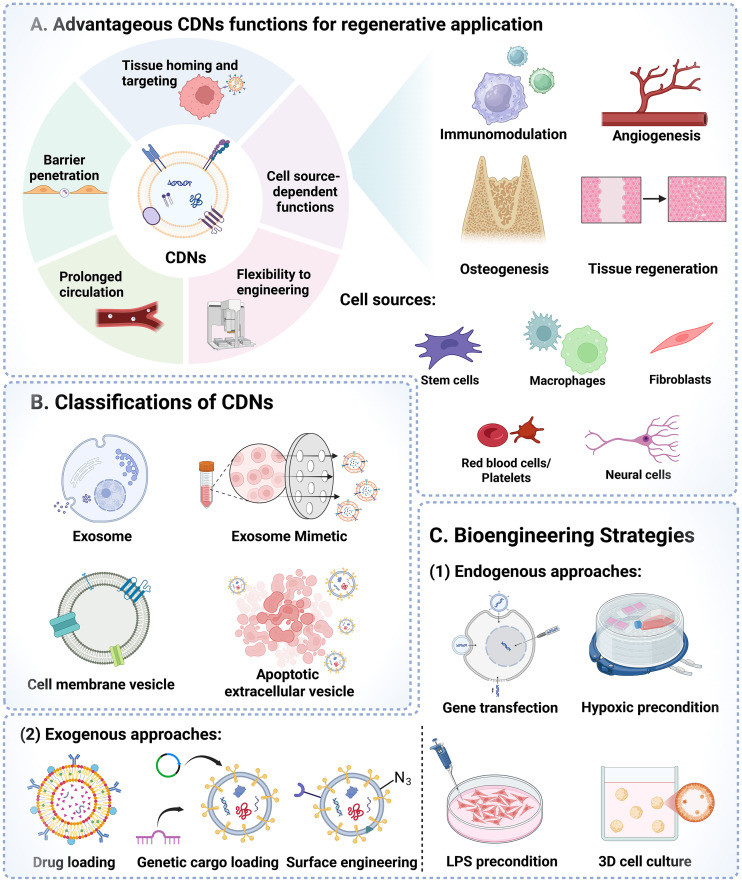
Schematic illustration of CDNs for regenerative application. (A) Advantageous functions and common cell sources: overview of the intrinsic properties of CDNs and their therapeutic functions in immunomodulation, angiogenesis, osteogenesis, and tissue regeneration, categorizing common cell sources used for CDN production. (B) Classifications: categorization of CDNs based on their origin and fabrication method, distinguishing between naturally secreted vesicles (exosomes, apoptotic extracellular vesicles) and engineered vesicles (exosome mimetics, cell membrane vesicles). (C) Bioengineering strategies: approaches to enhance CDN functionality are divided into: (1) endogenous approaches, where parent cells are modified before CDN isolation; and (2) exogenous approaches, where isolated vesicles are directly modified *via* drug/genetic cargo loading or surface engineering. Created with https://www.biorender.com.

Recently, cell derived nanovesicles (CDNs) have emerged as promising tools for tissue engineering with multiple advantageous biofunctions (Fig. 1). CDNs consist of a lipid bilayer encapsulating bioactive cargo, including nuclei acids, proteins, and metabolites, and exhibit extremely high biocompatibility with diverse biological functions. Importantly, CDNs retain key properties of their parent cells, conferring therapeutic effects analogous to cell-based therapies, such as intrinsic homing capability, induction of cellular differentiation, and robust immunomodulatory activity.^16^ On the other hand, CDNs bypass the significant safety risks associated with MSCs based therapy, such as potential tumor formation linked to their multipotency and self-renewal. Furthermore, CDNs are less susceptible to rapid clearance by the mononuclear phagocyte system.^17^ From a technical perspective, CDNs are more flexible to bioengineering modifications and can effectively penetrate biological barriers, thereby enhancing drug delivery with tissue-specific targeting. In the context of this review, CDNs is utilized as a broad umbrella term to encompass both naturally secreted vesicles and top-down engineered nanovesicles. Natural vesicles originate from cellular biogenesis (such as endosome-derived extracellular vesicles and apoptosis-derived vesicles), while engineered platforms utilize the native cellular component to enable the desired nanovesicles with higher yield (such as cell-derived lipid bilayers presenting native membrane proteins and exosome mimetics obtained through serial extrusion from whole cells). The rationale for grouping these distinct entities is their shared reliance on cell-derived lipid membranes with native surface receptors and bioactive cargos to evade immune clearance, transfer bioactive factors to recipient cells and mediate targeted tissue regeneration.

CDNs derived from a vast variety of cells have been investigated and applied for regenerative medicine (Fig. 1). Biofunctions of CDNs from different cell source have been widely explored, which are highly associated with the cell source from which it originated. Among them, MSCs are the most common cell source. It has been reported that MSCs showed therapeutic effects through paracrine functions of bioactive compounds, including cytokines, growth factors, metabolic products, and extracellular matrix components.^18^ And these secretory factors play major roles in regulations of migration, angiogenesis, and osteogenic differentiation. Specifically, bone marrow derived mesenchymal stem cells (BMSCs) are the mostly widely studied, which are the natural precursors of osteoblasts and their derived nanovesicles inherit signaling molecules primed for osteogenesis. Recent findings reveal that neonatal umbilical cord mesenchymal stem cell (UCMSC) is a source of extracellular vesicles, and could rejuvenate senescence of adult BMSCs, enhancing the regenerative capacities in bone formation, angiogenesis and wound healing.^19^ However, it is hard to harvest other types of MSCs in dental clinics, while dental MSCs emerge as a more promising cell source with minimal invasive and potential patient customized features, including dental pulp stem cells (DPSCs), periodontal ligament stem cells (PDLSCs) and dental follicle stem cells (DFSCs), *etc*.^20–22^ Moreover, gingival fibroblasts (GFs) also provide alternative options relevant to CDNs applied in oral diseases. GFs are dominate cell in gingival tissue that originate from mesenchyme with self-renewal and multi-differentiation capacities. Except for MSCs, immune cells are another alternative for cell source to derive CDNs due to their inflammatory and immunomodulation functions. Macrophages as dynamic cells transforming between two phenotypes, including pro-inflammatory M1-like macrophage and pro-reparative M2-like macrophage, participates in induction and resolution of inflammation.^23^ Cytokines secreted by M1-like macrophage facilitate immune activation and initiate the destruction mediators, including tumor necrosis factor alpha (TNF-α), interleukin-6 (IL-6) and interleukin-1 beta (IL-1β), *etc*. In contrast, M2-like macrophages secrete anti-inflammatory cytokines to suppress inflammation and restore homeostasis, such as transforming growth factor-β (TGF-β), interleukin-10 (IL-10) and vascular endothelial growth factor (VEGF), *etc*.^24^ The upregulated pro-reparative factors have been reported to play pivotal role in tissue regeneration, making M2-like macrophage a promising cell source to isolate CDNs in regenerative medicine. Recently, Schwann cells (SCs), the primary glial cells in the peripheral nervous system, have attracted growing attention in CDN isolation applied in regenerative medicine. Several studies have highlighted that SCs secrete neurotrophic factors to promote axonal myelination, facilitate the innervation of injured area, and accelerate tissue regeneration in a paracrine manner.^25,26^ Meanwhile, red blood cells (RBCs) and platelets are other sources of CDNs for prolonged circulation and targeting capacity towards injured or inflamed tissue due to specific membrane markers.^27,28^ The mature RBCs and platelets are the most abundant cells in the blood without cell nucleus, making them excellent choice for massive isolation of cell membranes.^29^ However, it is essential to emphasize that the functional inheritance of CDNs is strictly dependent on the biological properties of their parent cell source. Huang *et al.* demonstrated this cell-source dependency by comparing CDNs derived from adipose-derived stem cell (ADSC) and HEK293 cells, a functionally inert human embryonic kidney cell line widely used in biotechnology and pharmaceutical studies. The results showed that ADSC-derived CDNs exhibited cardioprotective effects in a model of myocardial ischemia-reperfusion, while CDNs generated from HEK293 cells did not confer protection.^30^

**Fig. 2:**
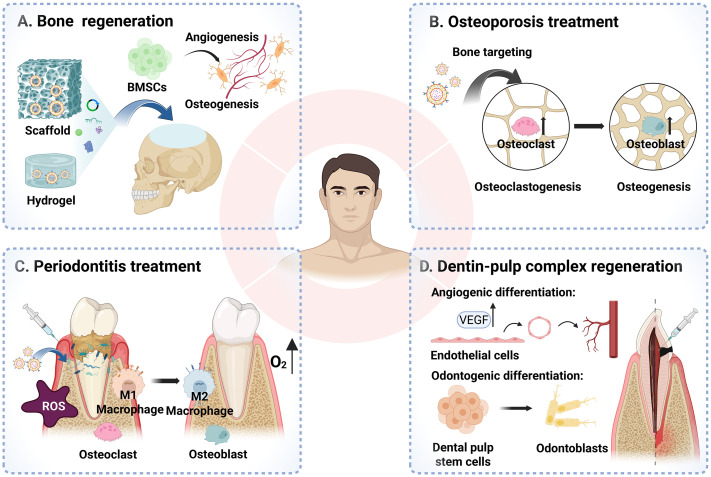
Therapeutic applications of CDNs in craniofacial tissue regeneration. (A) Bone regeneration: CDNs incorporated into biomaterial carriers recruit BMSCs and promote angiogenesis and osteogenesis to facilitate bone reconstruction. (B) Osteoporosis treatment: CDNs with bone-targeting properties are used to restore the balance of bone remodeling. (C) Periodontitis treatment: CDNs reduce ROS levels at the inflammatory sites, modulate the polarization of macrophages and improve new bone formation. (D) Dentin–pulp complex regeneration: CDNs stimulate the angiogenic differentiation of endothelial cells and induce the odontogenic differentiation to facilitate dentin–pulp complex regeneration. Created with https://www.biorender.com.

This review summarizes four major classes of cell-derived nanovesicles, outlining their advantages and limitations with respect to isolation, characterization, and distinct biological functions. By synthesizing evidence across craniofacial bone regeneration, osteoporosis, periodontitis, and dentin–pulp complex regeneration, we highlight the breadth of their therapeutic efficacy, and the underlying mechanisms involved (Fig. 2). Collectively, these findings emphasize the transformative potential of CDNs as a cell-free platform for craniofacial tissue engineering and their promise for future clinical translation in regenerative medicine.

## 2. Literature search strategy

To ensure a comprehensive and transparent review of the literature, an electronic database search was conducted across PubMed, Scopus, and Web of Science through March 2026. The following keywords were used individually and in combination: exosome, extracellular vesicle, exosome mimetic, membrane nanovesicle, apoptotic extracellular vesicle, apoptotic body, ApoEV, cell-derived nanovesicle, bone regeneration, tissue engineering, oral regeneration, craniofacial regeneration, osteoporosis, periodontal, periodontitis, alveolar bone, dental pulp, and maxillofacial. Only peer-reviewed articles published in English were considered. Articles were selected based on relevance to the topic, with priority given to studies published within the last five years. Clinical trial registries including ClinicalTrials.gov and the WHO International Clinical Trials Registry Platform (ICTRP) were also consulted to identify registered human trials.

## 3. Cell derived nanovesicles categories

### 3.1 Exosome

Exosomes are nanosized (30–150 nm) vesicles of endosomal origin which eukaryotic cells release into the extracellular environment.^31^ They serve as tools in intercellular communication by transferring membrane and cytosolic proteins, lipids and RNA to their recipient cells, resulting in altered functions of target cells.^32^ They have gained considerable research attention owing to native bioactive cargo with multiple biofunctions, which can be internalized through endocytosis or phagocytosis and transport specified information. However, a major challenge in the field remains the standardization of isolation and characterization methods. Although multiple techniques have been developed for exosome isolation, differential ultracentrifugation following cell culture remains the most widely used approach. This method sequentially removes cell debris, nuclei, and larger vesicles, followed by high-speed centrifugation at approximately 100 000*g* to pellet exosomes, yielding relatively high purity with minimal contamination. The high-speed and strong force of ultracentrifugation has been shown to increase vesicle aggregation.^33^ Challenged by the heterogeneity and small size of exosomes, and a lack of universal identification method, subsequent exosome characterization mostly relies on multiple measurement techniques, including quantification of particle size, quantification of total protein, characterization of morphology, and characterization of protein composition. Many studies detected specific biomarkers associated with vesicle biogenesis, although the functional roles of many markers require further investigation. Commonly used enrichment markers include tetraspanins (CD9, CD63, and CD81) and proteins related to the endosomal sorting complexes required for transport (ESCRT), such as tumor susceptibility gene 101 (TSG101) and apoptosis-linked gene 2-interacting protein X (ALIX).^34^ While many studies reviewed herein utilized the term ‘exosome’, it is important to note that according to MISEV guidelines, studies that does not prove biogenesis should be more accurately described as extracellular vesicles (EVs).^35^

**Fig. 3:**
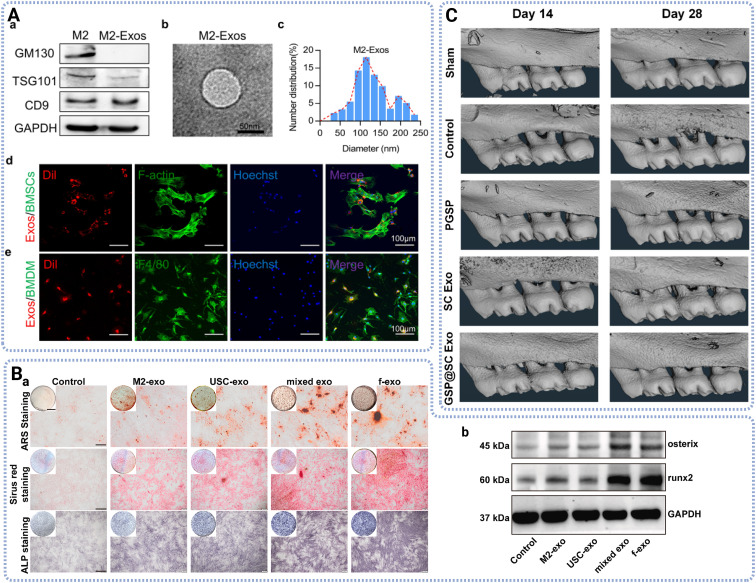
Characterization and therapeutic functions of exosomes. (A) Characterization of exosomes derived from M2-like macrophages, showing the surface markers, particle morphology, particle size distribution and cellular uptake by BMSCs and BMDMs. Adapted from ref. 40. Licensed under CC BY 4.0. (B) The regulation of osteoblast differentiation by fused exosomes (derived from M2 macrophage and urine-derived stem cell) *in vitro*. Adapted from ref. 41. Licensed under CC BY-NC-ND 4.0. (C) GSP@ Schwann cell exosome nanoparticles facilitated periodontal tissue regeneration, showing with representative micro-CT images at day 14 and day 28. Adapted with permission from ref. 50. Copyright 2025 American Chemical Society.

Exosomes derived from stem cells have been extensively investigated and demonstrate superior therapeutic potential in regenerative medicine as Fig. 3 displayed. Chew *et al.* reported that MSC-derived exosomes are rapidly internalized by periodontal ligament cells within minutes, trigger intracellular signaling within 15 minutes, and enhance cell migration and proliferation *via* CD73-mediated adenosine receptor activation of pro-survival AKT and ERK pathways.^36^ Qiao *et al.* reported that DPSC derived exosome promoted osteogenesis of PDLSCs and regulated inflammation by inhibiting the IL-6/JAK2/STAT3 signaling pathway *in vitro*.^37^ Albougha *et al.* reported that exosome from PDLSCs promoted osteogenesis in human osteoblast-like cells *in vitro* and improved new bone formation at the cranial bone defects in rats.^22^ While it has been reported that exosomes from human periodontal ligament cells (hPDLCs-Exos) promoted osteoclast differentiation associated with orthodontic tooth movements, enhanced M2 macrophage polarization, and inhibited pro-inflammatory M1 macrophage polarization.^38^ Ahmad *et al.* delivered a mechanistic review of PDLSC-Exos in the improvement of cellular proliferation, the enhanced osteogenesis and the modulation of inflammatory responses by regulating signaling pathways, resulting in a significant therapeutic efficacy in periodontal regeneration.^39^

However, macrophages derived exosomes also proved pivotal roles in tissue regeneration *via* inflammatory modulation. Chen *et al.* isolated exosomes from M2-like macrophages and identified their characterization with surface marker, particle size and cellular uptake by BMSCs.^40^ They found that M2 exosomes promoted BMSCs differentiation toward osteoblasts while suppressing bone marrow derived macrophage (BMDM) osteoclast formation *via* IL-10/IL-10R pathway activation, reducing alveolar bone resorption in mice with periodontitis *in vivo*.^40^ Li *et al.* presented that exosomes derived from M2 macrophages inhibited adipogenesis and promoted osteogenesis of BMCS through miR-690/IRS-1/TAZ axis. Currently, fusion vesicles have gained attention in regenerative medicine due to their dual therapeutic effects from individual components. Ma *et al.* utilized co-extrusion technique to fabricate fused exosomes with MSC-exo and M2-exo, which precisely targeted the inflammatory sites, promoted angiogenesis, and improved osteogenesis while inhibited osteoclast activity.^41^ Additionally, SC derived exosomes have been shown to promote innervation, immunoregulation and vascularization, leading to enhanced bone regeneration *in vivo* by upregulating the TGF-β1/SMAD2/3 signaling pathway.^42^ And Cui *et al.* reported that SC Exo induced osteogenic and neurogenic differentiation of hPDLCs, promoted the neurogenesis of periodontal tissue, and accelerated periodontal bone regeneration in a rat model.^43^

Bioengineering strategies have been applied at cellular and molecular levels to enable exosomes more biofunctions and to achieve better performances, mainly divided into endogenous and exogenous engineering methods. Exogenous engineering methods have been used to load therapeutic cargos and modify exosome surface to increase specific tissue targeting. Among these encapsulated cargos, miRNA have attracted significant attention for their regulatory roles in various biological process modulation.^44^ Several studies have employed MSC derived exosomes to load crucial miRNAs to promote bone and periodontal regeneration, including miR-26a, miR-935 and miR-222, *etc*.^45–47^

In contrast, endogenous approaches often involve biological pre-treatment with parent cells to deliver bioactive cargo without adverse effect on the structural integrity. Gene transfection is a common pre-treatment on donor cells to display therapeutic nuclei acid cargos. Li *et al.* highlighted that bone morphogenetic protein-2 (BMP-2) genetically engineered MSC through liposome-mediated gene transfection could produce MSC-BMP2-Exo, which significantly promoted bone regeneration in mouse femur defect model.^48^ This effect was attributed to synergistic effect of the natural contents of exosomes and the up-regulated BMP2 expression, indicating that MSC derived exosome could serve as regenerative agents and efficient gene delivery platform. Additionally, microRNAs (miRNAs) play pivotal roles in inflammatory signaling and MSC differentiation during regenerative processes. Qiu *et al.* showed that exosome derived from miR-150-3p transfected BMSCs promoted osteoblast proliferation and differentiation, and inhibited apoptosis compared to exosome derived from non-treated BMSCs.^49^ Moreover, lipopolysaccharide (LPS) preconditioned DFSC derived exosomes, which enhanced the proliferation, migration and differentiation of PDLSCs *in vitro*. *In vivo*, these enriched exosomes repaired lost alveolar bone at the early stage and maintain the level of it later in experimental periodontitis rats, resulting in decreased expression of the RANKL/OPG ratio.^21^

### 3.2 Exosome mimetics

**Fig. 4:**
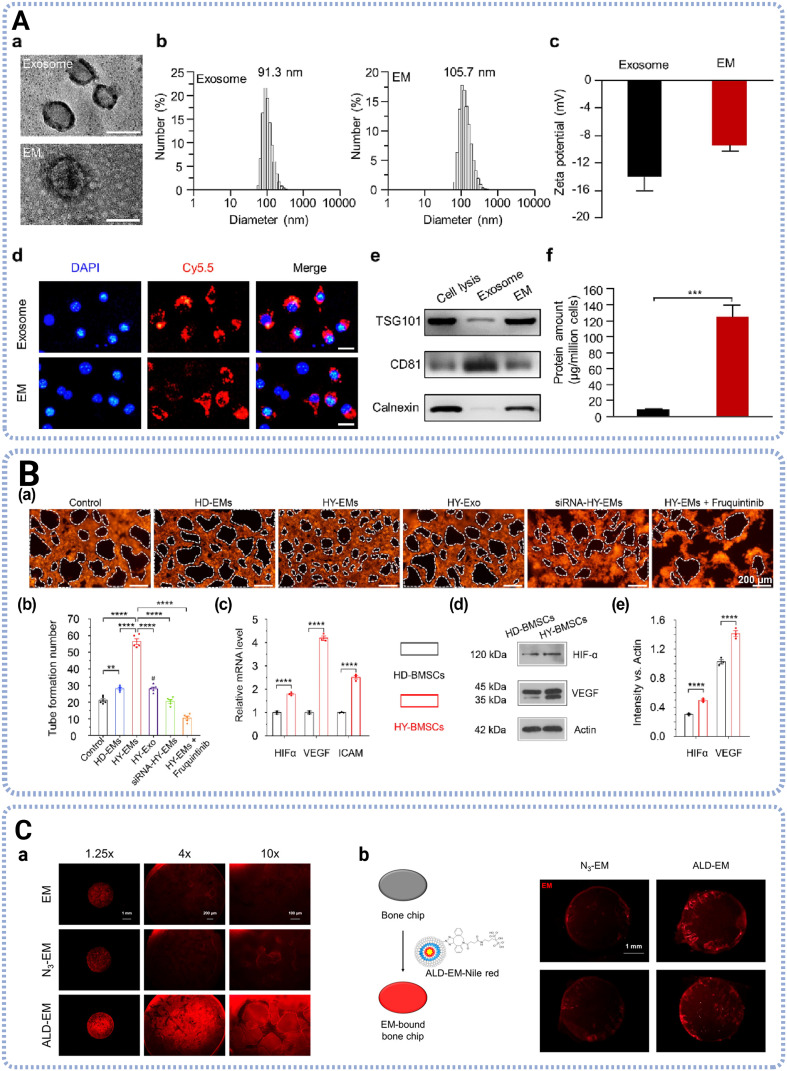
Characterization and therapeutic functions of EMs. (A) Characterization of exosomes and EMs, showing the particle morphology, size distribution, zeta potential, cellular uptake, surface markers and yield of protein concentration of both exosomes and EMs. Adapted with permission from ref. 60. Copyright 2023 Elsevier. (B) EMs derived from BMSCs pretreated with hypoxia improved angiogenesis *in vitro*, showing representative image of tube formation and expression of angiogenic genes and proteins. Adapted with permission from ref. 56. Copyright 2024 Elsevier. (C) The binding ability of modified EMs on the HA-coated PLGA scaffold and mouse skull bone. Adapted with permission from ref. 61. Copyright 2023 American Chemical Society.

While exosomes inherently exhibit therapeutic effects, the low yield restricts their board application and makes it challenging to obtain sufficient source for further functional modification. Exosome mimetics (EMs) have emerged as a promising alternative to exosomes, containing bioactive cargo with significantly higher yield. Briefly, cell suspensions are mechanically extruded to sequentially pass through serial polycarbonate membrane filters with specific size (5 μm, 1 μm, 400 nm, *etc*.) to isolate EMs. Except for higher production yield, EMs also exhibit different components and therapeutic effects compared to exosomes as Fig. 4 displayed. In a previous study, Zhang *et al.* identified 2325 proteins from exosomes and EMs, both derived from UCMSCs, and results showed that 1669 proteins shared with exosomes and EMs played roles in retrograde vesicle-mediated transport and vesicle budding from the membrane. While the 264 proteins unique to EMs targeted the cell membrane, the 382 proteins unique to exosomes participated in extracellular structure and matrix organization.^51^ A proteomics analysis revealed that EMs derived from human UCMSCs significantly enriched mitochondrial-derived oxidative phosphorylation-related proteins in comparison to hUCMSCs derived exosomes, leading to promoted skin wound healing when loaded in gelatin methacryloyl (GelMA) hydrogels.^52^

Similar to exosomes, bioengineering strategies have been employed to endow EMs with desired biological functions. In biomedical applications, these approaches primarily involve either cargo modulation through exogenous loading or donor-cell pretreatment, or the functionalization of EMs *via* surface engineering. Regarding exogenous cargo loading, Zha *et al.* reported that EMs derived from ATDC5, a chondrogenic progenitor cell line, loaded with the plasmid of VEGF, promoted angiogenesis *in vitro* and induced vascularized osteogenesis *in vivo*.^53^ Zhang *et al.* developed EMs from pluripotent stem cells-derived endothelial cells to deliver Dapagliflozin, a sodium-glucose linked transporter 2 (SGLT2) inhibitor, promoting neovascularization *via* HIF-1α/VEGFA pathway.^54^ And Wang *et al.* demonstrated that BMSC-derived EMs serve as a superior biological nanocarrier for doxorubicin, which exhibited high delivery specificity to osteosarcoma tissue and exerted potent anti-tumor effects.^55^

Additionally, pre-treatments have been applied to parent cells to fabricate EMs with desired biofunctions. Zhong *et al.* proved that EMs derived from BMSCs pretreated with hypoxia significantly improved the tube number of human umbilical vein endothelial cells (HUVECs) *in vitro*, and promoted vascularized osteogenesis with the new bone volume and vessel number in new bone increased to 120% and 175%, respectively.^56^ Cui *et al.* reported that EMs prepared from human induced pluripotent stem cells-derived endothelial cells under hypoxia culture could enrich abundant membrane C–X–C motif chemokine receptor 4, enabling their endothelial homology and facilitating the bone marrow endothelial cells (BMECs) tropism.^57^ Xu *et al.* revealed that EMs produced from MSCs cultured in three-dimensional (3D) cell culture systems with osteogenic differentiation medium enhanced bone tissue regeneration related to Wnt/β-catenin and Notch signaling pathways.^58^ Jiang *et al.* developed EMs from simvastatin (SIM)-pretreated MSCs and demonstrated that SIM-MSCs-EM exhibited excellent angiogenesis and osteogenesis regulation due to endogenous miR-29b-3p cargo to silence gene phosphatase and tensin homolog (PTEN) and activate the PI3K/AKT pathway.^59^

Various strategies have been applied for EMs surface engineering, including covalent modification hydrophobic interaction, and receptor–ligand interaction. Covalent modification forms a stable covalent bond between functional molecules and EMs surface components, and particularly copper-free click chemistry reaction offer a versatile option to modify EMs with high efficiency and specificity, while preserving structure and function of EMs. Specifically, Hao *et al.* found that MSC derived EMs modified with bone targeting peptide aspartic acid 8 (Asp8), showing high affinity for hydroxyapatite, and subsequently loaded with miR-26a, a Wnt signaling pathway activator, significantly promoted bone regeneration in both femur defect and osteoporosis disease models.^60^ And Lee *et al.* modified the MCS EMs with alendronate (ALD), a bone targeting ligand, with dibenzocyclooctyne (DBCO) as the clickable material with azide, and confirmed a high bone affinity with a mouse skull bone chip *in vitro*.^61^ Interestingly, Lu *et al.* developed Cx43-enriched EMs by integrating connexin 43 (Cx43) into synthetic lipid bilayers using a cell-free protein synthesis system. These small interfering (siRNA)-loaded nanoparticles facilitate targeted delivery into Cx43-expressing recipient cells, leveraging the protein's natural channel-forming capabilities.^62^ Additionally, Zha *et al.* employed Biotin–Avidin-System (BAS) to immobilize VEGF plasmid loaded EMs onto chitosan (CS)/poly(lactic acid) (PLA) nanofiber film to treat rat cranial defects, showing good physical absorption and covalent binding integration.^53^

### 3.3 Cell membrane nanovesicles

Cell membrane nanovesicles is an excellent drug delivery system due to its complex cues directly inherited from native cell membranes, evading the surveillance of the immune system and homing to the specific targeting tissue. Various methods can be used for cell membrane isolation, such as hypotonic solution, freezing and thawing circulation, and ultrasonic disruption, followed by low-speed centrifuge to remove the organelles and centrifuge with ultra-high speed to collect supernatant.^63^ Obtained cell membranes are characterized by the functional surface markers. For example, CD47, a “don't eat me” signal, prevents immune clearance and leads to prolonged circulation time.^64^ C–X–C chemokine receptor type 4 (CXCR4), a receptor of chemo-attractant stromal cell-derived factor 1 (SDF-1), facilitates cell derived membrane accumulating in bone microenvironments with functional chemokine-mediated tissue targeting.^65^

So far, several drugs have been reported to be efficiently carried and delivered by cell derived membrane in regenerative studies. Different cell derived membranes possess different characteristics for particle platforms, sourced from macrophages, platelets, BMSCs, *etc*., due to their high biocompatibility, pharmacokinetic properties, and specific tissue as well as disease targeting.^66,67^ Deng *et al.* genetically engineered toll-like receptors 4 (TLR4)-expressing RAW264.7 macrophages, isolated their membranes, and coated minocycline loaded silk fibroin nanoparticles to prepare biomimetic nanoparticles (MSNCs).^68^ As TLR4, a predominant bacterial endotoxin receptor, activated the innate immune response, MSNCs effectively targeted bacteria, neutralized LPS in the microenvironment and alleviated inflammation induced bone loss *in vivo*.^68^ Qu *et al.* fabricated a biomimetic nanomodulator using polyphenolic antioxidants and flavonoid quercetin coated with macrophage membranes to specifically target and polarize M1 macrophages toward anti-inflammatory M2 subtype.^69^

**Fig. 5:**
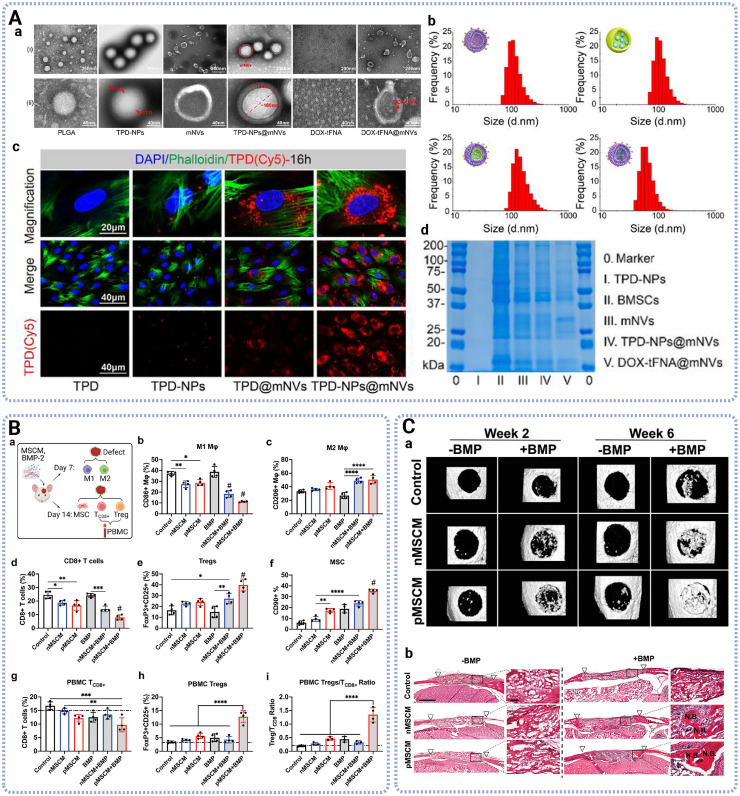
Characterization and therapeutic functions of cell membrane nanovesicles. (A) Characterization of BMSC derived membrane coated nanoparticles, showing the particle morphology, size distribution, cellular uptake by BMSCs and surface markers. Adapted with permission from ref. 74. Copyright 2025 Wiley. (B) BMSC derived membrane coating synergizes with BMP-2 modulated both innate and adaptive immune responses and recruited stem cells *in vivo*. Adapted with permission from ref. 75. Copyright 2023 Wiley. (C) BMSC derived membrane coating synergizes with BMP-2 enhanced bone regeneration *in vivo*, showing representative CT images and Hematoxylin and Eosin (H&E) staining images. Adapted with permission from ref. 75. Copyright 2023 Wiley.

Cell membrane has also been applied to coat nanoparticles to fabricate membrane-coated nanoparticles which are inherently multifunctional, combining the bio-interfacing properties of cell membranes with the synthetic properties of nanoparticle cores.^70^ Compared with cell derived extracellular vesicles, nanoparticles shaped by cell membranes exhibited extensive advantages, including the quantity able to be produced, their high purity, and the ability to conveniently manipulate them by encapsulating the core cargo with co-extrusion.^71^ Shi *et al.* reported that macrophage membrane-coated nanoparticles containing osteoconductive Ca_3_(PO_4_)_2_ and bactericidal TiO_2_ showed superior recognition and adsorption with bacteria, toxins and inflammatory cytokines, demonstrating enhanced anti-inflammation and anti-bacterial properties.^72^ Wang *et al.* developed platelet membrane coated hepatocyte growth factor-poly(lactic-*co*-glycolic acid) (PLGA) nanoparticles (NPs), enhancing endothelial cell proliferation, migration, and tube formation *in vitro*, where platelets membrane leveraged the natural homing ability to vascular injury sites *in vivo*.^73^ As shown in Fig. 5, Ma *et al.* highlighted that engineered membrane nanovesicles deliver the core of teriparatide-loaded PLGA were able to target bone tissue, significantly slow bone loss and alleviate osteoporosis indicators *via* systemic intravenous (IV) injection administration.^74^ Su *et al.* demonstrated that MSC membrane coating on scaffolds promoted macrophage polarization toward regenerative phenotype, induced CD8+ T cell apoptosis, and enhanced regulatory T cell differentiation, leading to accelerated bone regeneration and suppressed inflammation when combined with a low dosage of BMP-2 ^75^ (Fig. 5).

### 3.4 Apoptotic extracellular vesicles

Apoptosis plays an important role in physiological homeostasis management, and apoptotic extracellular vesicles (ApoEVs) are extracellular vesicles released from cells during apoptosis.^76,77^ Current evidence suggests that they originate through exosome-like biogenesis pathways, and indicates that they contain complex cargos including mitochondria, DNA, RNAs, proteins and ribosomes, programming cell fate in both physiology and pathological scenarios.^78^ Even though the knowledge of ApoEV lags behind that of exosomes, their communication roles between cells are increasingly recognized, demonstrating therapeutic potentials in tissue regeneration.^79^ In contrast to exosomes, the diameters of ApoEVs range from 50 to 5000 nm, and could be further classified into three subtypes according to their biogenesis and size: apoptotic body (ApoBDs, 1–5 μm in diameter), microvesicle-like ApoEVs (ApoMVs, 100–1000 nm in diameter), and exosome-like ApoEVs (ApoExos, <150 nm in diameter).^80^ Various subtypes not only vary in size but also express different surface proteins and exhibit different characteristics.^81^ However, achieving separation of these subtypes remains technical challenges, as their size range and physical properties frequently overlap with each other. As a result, current isolation protocols yield enriched ApoEV subpopulations rather than pure ApoEVs, underscore the critical need for high-resolution separation techniques in further investigations.

**Fig. 6:**
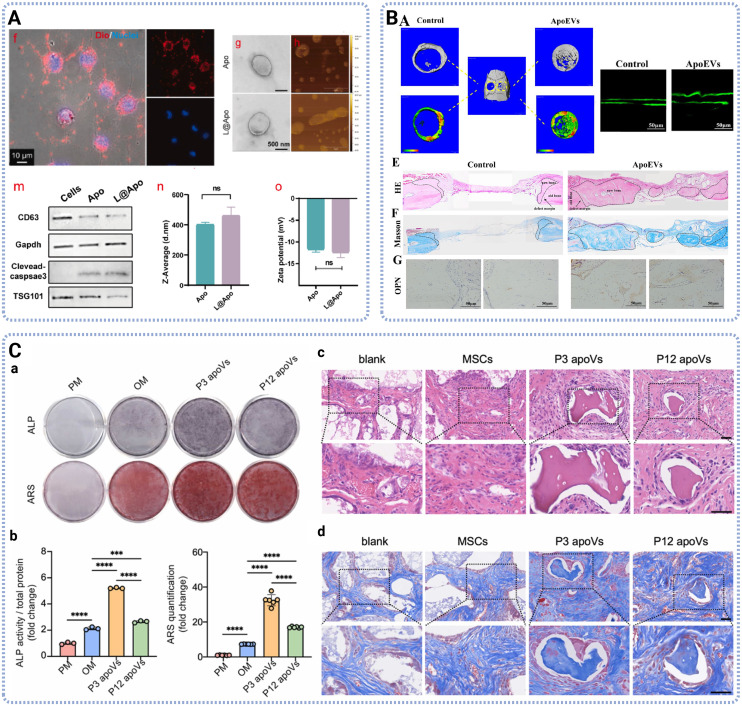
Characterization and therapeutic functions of ApoEVs. (A) Characterization of ApoEVs isolated from BMSC treated with LAPONITE®, showing ApoEVs and mitochondria colocalization, TEM and AFM images, surface markers, average size and zeta potentials. Adapted with permission from ref. 94. Licensed under CC BY-NC-ND 4.0. (B) BMSC-derived ApoEVs promoted bone repair in the rat calvarial bone defect model, showing the representative CT images, bone formation rate, H&E and Masson staining images. Adapted with permission from ref. 92. Copyright 2022 SAGE Publications. (C) Osteoinductive capacity of MSC-derived ApoEVs *in vitro* and *in vivo*, showing the ALP and Alizarin Red S (ARS) images *in vitro*, H&E and Masson staining *in vivo*. Adapted with permission from ref. 86. Copyright 2026 Elsevier.

Due to their phagocytic targeting and abundant functional cargos, ApoEVs demonstrate great therapeutic effectiveness in immunomodulation, homeostasis restoration, and angiogenesis, which are essential processes in subsequent bone tissue regeneration as Fig. 6 displayed. Li *et al.* reported that ADSC-derived ApoEVs abundantly expressing miR-21-5p induced Ana-1 macrophages toward M2 phenotype and improved the angiogenesis of endothelia cells.^82^ Calreticulin on the BMSC-ApoEVs surface expressed ‘eat me’ signals, attracted phagocytosis by hepatic macrophages, and restored macrophage homeostasis, leading to inhibition of macrophage accumulation and transformation toward anti-inflammation phenotype.^83^ Xin *et al.* reported that hUCMSC derived ApoEVs suppressed proinflammatory gene expression (*IL-1β*, *TNFα*, *IL-6*, and *IFN-γ*), while triggering the expression of neovascularization genes (*IL-10*, *VEGFA*, *IGF1*, and *TGFβ*) in primary macrophages, which were associated with the enhancement of mitochondrial bioenergetics of recipient cells.^84^

Furthermore, ApoEVs derived from various MSCs have been shown to promote osteogenesis and bone regeneration through distinct underlying mechanisms. Li *et al.* BMSC-ApoEVs were easily phagocytized by endogenous BMSCs and promoted their migration, proliferation and osteogenic differentiation, by transporting Ras protein to activate the Ras/Raf1/Mek/Erk pathway.^85^ Jiang *et al.* identified integrin α 10 (ITGA10) as a highly efficient osteoinductive agent in MSC-ApoEVs and fabricated ITGA10-enriched ApoEVs regulating MSC osteogenesis *via* AKT signaling activation.^86^ Yu *et al.* demonstrated that MSC-derived ApoEVs were internalized by vascular endothelial cells, where they preferentially localized to the endoplasmic reticulum. The enrichment of oxidized phosphatidylcholine in these vesicles induced endoplasmic reticulum stress, which upregulated adhesion molecule expression and thereby promoted the recruitment of autologous stem cells from the vasculature to the defect site, enhancing osteogenesis and bone regeneration.^87^ Regarding dental stem cells, Yang *et al.* isolated DPSC-ApoEVs and found that they could enhance bone mineral density and trabecular bone count in mouse OVX model by activating the extracellular regulated kinase (ERK)1/2 signaling pathway.^20^

Beyond the extensively studied mesenchymal stem cell sources, other cell types have also been utilized for the isolation of ApoEVs. Mature RBCs, which lack of nuclei and genetic material, offer high biological safety and are readily obtainable as a source of ApoEVs. Shao *et al.* investigated the role of RBC-ApoEVs in osteogenesis of hBMSCs, and it showed that RBC-ApoEVs promoted osteogenesis by transferring anhydrase 1 (CA1) and activating the P38 MAPK pathway.^88^ And Jiang *et al.* utilized platelets (PLT) as a source of ApoEVs, as large quantities of platelets can be readily obtained from whole blood donations. Because platelets lack nuclei, safety concerns related to genetic material are minimized, and their isolation does not require *in vitro* culture or growth media, thereby improving cost-effectiveness and reducing the risk of contamination. The results showed that platelets could rapidly release abundant ApoEVs, and six proteins specifically enriched in PLT-ApoEVs, including CD41, CD61, CD62P, ACKR2, SEZ6L2, and GOLPH2.^89^

In addition, studies have shown that pretreatment of parent cells had an impact on the therapeutic effects of ApoEVs. Ding *et al.* studied ApoEVs derived from ADSC cultured under hypoxia, which significantly promoted endogenous stem cell migration, polarized BMDMs towards M2 phenotype, and accelerated cartilage repair *in vivo*.^90^ Compared to normoxic and hypoxic MSC-ApoEVs, oxidatively stressed MSC-ApoEVs were more effective in angiogenesis promotion, probably due to its intrinsic cargo miR-210-3p thereby activating AKT signaling.^91^ Interestingly, ApoEVs generated from different stages demonstrated different therapeutic capacities and regenerative potentials. Li *et al.* found that ApoEVs derived from cell in the middle stage of apoptosis mostly enhanced the regenerative capacity of BMSCs, by increasing intracellular ROS to activate JNK signaling.^92^ Similarly, Ma *et al.* reported that ApoEVs from mononuclear preosteoclasts mainly promoted endothelial progenitor cell differentiation, whereas mature osteoclasts derived ApoEVs facilitated osteogenic differentiation *via* RANKL reverse signaling.^93^ Besides, BMSCs treated with LAPONITE®, lithium magnesium sodium silicate, generated engineered ApoEVs, which eliminated dysfunctional mitochondria while initiating biogenesis to reconcile the high adenosine triphosphate (ATP) demands of osteogenesis, *via* PINK1/Parkin-mediated mitophagy activation.^94^

### 3.5 Similarity and difference among the CDNs

While the previously mentioned CDNs all exhibit therapeutic potential for oral and craniofacial tissue regeneration, particularly in promoting immunomodulation, angiogenesis and osteogenesis, and can be tailored *via* bioengineering strategies, they present distinct biological features and specific translational trade-offs. Table 1 summarizes the core characteristics and unique features of these four types of CDNs based on their origins and fabrication techniques, comparing their respective advantages and limitations in the context of current regenerative medicine research.

**Table 1:** The distinct characteristics of CDNs in oral and craniofacial tissue regeneration

CDN type	Size range	Core characteristics	Scalability & yield	Typical purity/contaminants	Unique features	Advantages (Pros)	Limitations (Cons)
Exosome	30–150 nm (ref. 31)	Naturally secreted vesicles mediating intercellular communication	Low to moderate	Moderate to high; non-vesicular proteins and lipoproteins	Endogenous cargo possessing diverse biological functions	High therapeutic potential; excellent natural drug delivery systems	Low yield; lack of standardized isolation and characterization protocols
EM	Controlled size, depending on pore size of membranes during extrusion	Nanovesicles generated *via* serial mechanical extrusion of cells	High	Low; contaminated by randomly encapsulated nuclear and cellular organelle debris	Fabrication utilizing bioengineering strategies	High yield; tunable particle size; readily modified during extrusion	Contains a heterogeneous mixture of intracellular components
mNV	100–300 nm (ref. 95)	Derived directly from isolated native cell membranes	High	High; potentially contaminated by residual cytoskeletal proteins and organelle membranes	Inherent tissue targeting and homing abilities based on source cell lineage	Facilitates systemic administration (*e.g.*, *via* biomimetic coating) by specific tissue targeting	Lacks the complex intracellular therapeutic cargo found in naturally secreted vesicles
ApoEV	50 nm–5 µm (ref. 81)	Extracellular vesicles naturally released during programmed cell death	Low to moderate	Highly heterogeneous; containing nuclear chromatin and organelle fragments from apoptosis	Highly attractive to phagocytes, leading to enhanced cellular internalization	Superior therapeutic potential; excellent for restoring tissue homeostasis	Subtypes are not thoroughly characterized; impacts vary based on the stage of apoptosis

## 4. The application of engineered CDNs in craniofacial tissue regeneration

Although the beneficial roles of CDN cargos in regulating the craniofacial microenvironment have been increasingly recognized, their limited yield and inherent functional constraints still result in suboptimal therapeutic efficacy for meeting the regenerative demands of both soft and hard tissues. Engineering nanovesicles is the process of modifying and optimizing nanovesicles’ surface or cargo, which enables multiple biofunctions or improves their specific recognizing, targeting and drug delivery to recipient cells or tissue. Herein, we mainly focus on four clinical scenarios characterized by high prevalence and significant needs for regeneration intervention, including craniofacial bone defects, systemic osteoporosis, periodontitis and dental pulp necrosis. To guide future clinical translation, Table 2 outlines a design framework for these conditions, mapping the preferred CDN platform, required engineering levers and target therapeutic outcomes.

**Table 2:** Translational design framework for CDN-based therapies

Clinical scenario	Preferred CDN platform	Engineering levers	Target outcomes & overcoming barriers
Craniofacial bone defects	All four types reported	Loading osteogenic factors; surface modification with bone targeting capacity; cellular pretreatment; ECM-mimetic hydrogel embedding/immobilized on functional scaffolds	Sustained release of osteogenic factors; enhanced osteogenesis and angiogenesis; immunomodulation
Systemic osteoporosis	Mainly mNVs or membrane coated nanoparticles; exosomes, EMs and ApoEVs also reported	Surface conjugation with bone-targeting ligands; loading anti-osteoclastic factors; cellular pretreatment	Systemic delivery: evading immune clearance and homing to resorptive bone tissue; inhibited osteoclastogenesis
Periodontitis	All four types reported	Loading anti-inflammatory factors; surface modification with tissue targeting and inflammatory factor neutralization capabilities; cellular pretreatment	Immunomodulation; ROS clearance; anti-bacteria; homeostasis restoration; enhanced angiogenesis and osteogenesis
Dental pulp necrosis	Exosomes, membrane coated nanoparticles, and ApoEVs	Loading pro-angiogenic cargo; integrated with mineralized biomaterials for dentin bridge development	Promoted angiogenesis, neovascularization and dentinogenesis

### 4.1 Bone regeneration

Bone regeneration is a complex physiological process, involving diverse cell components, cytokines, growth factors and intercellular signaling pathways.^96^ While bone tissue processes an innate ability for self-repair, critical-sized bone defects exceed this regenerative threshold and require additional clinical interventions.^97^ To bridge these gaps, bone tissue engineering, particularly scaffolds functionalized with stem cells and growth factors, have gained extensive attention in bone regeneration. However, accumulating evidence showed that CDNs could act as superior alternatives to stem cell therapy with improved efficacy and less safety concerns.

Owing to the inheritance of parental cell surface markers and receptors, CDNs derived from different source cells exhibit intrinsic tropism for bone tissue. These vesicles selectively interact with key cellular components of the bone microenvironment, including BMSCs, osteoblasts, and osteoclasts, enabling targeted delivery of bioactive cargo. Such interactions precisely modulate recipient cell signaling and accelerate the regenerative cascade. Du *et al.* demonstrated that erythrocyte membrane-coated zeolitic imidazolate framework-8 (ZIF-8) nanoparticles loaded with cell division cycle protein 20 (CDC20) enhanced nanoparticle stability and promoted membrane fusion, thereby amplifying osteogenic differentiation of BMSCs and improving bone regeneration in cranial and subchondral defect models.^98^

The encapsulated bioactive cargos of CDNs have garnered significant attention for their ability to modulate gene expression and orchestrate the complex signaling cascades essential for bone regeneration. By delivering a diverse payload of nucleic acids, proteins and drugs, these vesicles act as potent mediators that drive the biological programs required for bone repair. Deng *et al.* developed GelMA incorporating DPLSC-ApoEVs and transplanted them in rat cranial defects, which could be endocytosed by endogenous BMSCs to facilitate their osteogenic differentiation and bone regeneration, by voltage dependent anion channel protein 1 (VDAC1) enriched in ApoEVs as an autophagy activator.^99^ A previous study showed that ApoEVs derived from cells in the middle stage of apoptosis when incorporated into GelMA composite implants, significantly improved new bone formation in calvarial defect.^92^ Cheng *et al.* studied both BMSCs and ADSCs derived Apo-EVs, and reported that compared to MSC-exosomes, MSC-ApoEVs specifically expressed hsa-miR-4485-3p with the greatest abundance and highest stability, while it served as a brake for osteogenesis, instead of an accelerator, participating in balancing bone metabolism.^100^ Subsequently, they generated tailored ApoEVs with downregulated hsa-miR-4485-3p, which exhibited significant osteoinductive effects and promoted bone formation in rat calvarial defects.^100^ Ma *et al.* reported that exosomes derived from BMSCs immobilized on 3D printed hydroxyapatite (HA) scaffolds demonstrated substantially upregulated expression of osteogenic-related genes and proteins, leading to satisfactory bone integration in cranial defects.^101^ Notably, Wu *et al.* found that exosomes secreted by stem cells from human exfoliated deciduous teeth upregulated the expression of angiogenesis and osteogenesis related genes *in vitro*, and facilitated alveolar bone regeneration by promoting neovascularization through the AMPK signaling pathway.^102^ Additionally, the incorporation of smoothened agonist (SAG), a small molecule drug, into EMs greatly improved the osteogenic property through the activation of hedgehog signaling, resulting in the enrichment of the osteogenic differentiation of the MSCs *in vitro* and the significantly enhanced reossification *in vivo*.^61^

Despite the significantly regenerative potentials of CDNs in bone regeneration, bioengineered CDNs provide a more robust therapeutic strategy in more challenging healing scenarios. Their ability to specifically target and rejuvenate diabetic or aging bone tissue makes them indispensable for clinical cases characterized by compromised regenerative capacity. For example, pretreating BMSCs with BMP2 generated specialized exosomes (BMP2-Exos) capable of modulating the immune response and reversing diabetic-induced osteogenic impairment. BMP2-Exos promoted M2 macrophage polarization and stimulated osteogenesis in diabetic BMSCs *via* the upregulation of PI3K/AKT signaling, which substantially enhanced cranial bone reconstruction within a diabetic defect model after it was integrated into a GelMA hydrogel delivery system.^103^ Surprisingly, exosomes derived from the serum of juvenile mice during bone fracture healing could rescue H_2_O_2_ induced cell senescence and promoted osteogenic process. Intriguingly, exosomes isolated from the serum of juvenile mice during fracture healing have been shown to rescue cells from H_2_O_2_-induced senescence and stimulate osteogenic differentiation.^104^ By functionalizing these vesicles with the bone-targeting peptide DSS6, a 6-repeat Asp-Ser-Ser sequence, these exosomes demonstrated superior efficacy in fracture healing in aged mice, driven by the activation of the Tomm7-dependent Pink1/Parkin mitophagy pathway, which restored mitochondrial homeostasis and effectively reversed the aging microenvironment in bone tissue.^104^

Encouragingly, emerging clinical trials have begun to validate the therapeutic application of CDNs for human bone regeneration, which mark a shift from bench-side research to clinical application, confirming the safety and osteoinductive capacity of cell-free therapies. A recent clinical study showed that Jiang *et al.* conducted a double-blind, randomized self-controlled trial to evaluate both the efficacy and safety of lyophilized MSC-ApoEVs for hemostasis and bone regeneration in patients with impacted third molar extraction. Excluding the 4 patients lost to follow up, all 39 patients tolerated MSC-ApoEVs well, which significantly shortened hemostatic time and facilitated alveolar bone regeneration.^105^

In addition, a clinical trial (ClinicalTrials.gov identifier: NCT04998058) is underway, investigating the efficacy of conditioned medium (CM) collecting from hADSCs culture for maxillary sinus augmentation. In this study, autologous hADSCs are harvested from abdominal lipoaspirates and CM is combined with a synthetic bone substitute as maxillary sinus graft. After 6 months, implants are placed, and regenerative outcomes are evaluated by bone biopsies from drilled implant site and volumetric assessment using cone beam computed tomography (CBCT). And commercial gelatin sponge-loaded ApoEVs were applied to evaluate the efficacy and safety of alveolar bone regeneration in patients with mandibular third molar extraction (ClinicalTrials.gov identifier: NCT05971342). Researchers (ClinicalTrials.gov identifier: NCT07508033) compared the bone regenerative potentials of MSC-derived exosomes and the currently clinically used platelet-rich fibrin on patients with tooth extraction after three months by CBCT.

### 4.2 Osteoporosis treatment

Osteoporosis is the most common metabolic bone disease, characterized by low bone mass, disruption of bone architecture, and deterioration of bone tissue, which increases the risk of fracture in patients.^106^ There is an imbalance between bone formation and bone resorption, where the expression of receptor activator of nuclear factor-κB ligand (RANKL) is increase and its decoy receptor osteoprotegerin (OPG) is downregulated, leading to excessive activation of receptor activator of nuclear factor-κB (RANK) on osteoclast membrane.^107^

A primary therapeutic goal in osteoporosis is to suppress excessive osteoclast activity. Accordingly, it is crucial for CDNs to possess bone-targeting and bone-binding capabilities, as they are typically administered systemically and off-target distribution should be minimized. Zhang *et al.* coated PLGA-MSC secretome NPs with cell membranes derived from CXCR4-expressing human microvascular endothelial cells, which inhibited osteoclast differentiation and promoted osteogenic proliferation *in vitro*, accumulating in bone and reducing ovariectomy (OVX)-induced bone mass attenuation in rats.^108^ And Cui *et al.* isolated the membranes from RANK and CXCR4 overexpressed BMSCs and coated these membranes on pH-sensitive chitosan-based nanogels containing synthetic parathyroid hormone 1-34 (PTH 1-34). The results showed that engineered membrane coated nanogels confer bone targeting ability, scavenge RANKL and responsively released therapeutic PTH 1-34 in bone tissue and exhibited reduced bone loss in OVX mouse model.^109^ Similarly, Chen *et al.* utilized Golgi glycoprotein 1 (GLG1) abundantly expressed cell membranes with bone tissue targeting to coat biomimetic mitochondrial minerals, which enhanced osteogenesis and angiogenesis by supplying phosphate to aid ATP formation, regulating cellular energy metabolism at osteoporotic bone sites.^110^ A previous study has shown that osteoclast derived ApoEVs (OC-ApoEVs) played a significant role in physiological bone turnover, which promoted osteogenesis by activating the downstream mTOR pathway *via* RANKL-mediated reverse signaling.^111^ Moreover, systemic administration of exogenous OC-ApoEVs effectively delayed the bone loss in OVX mice, validating that OC-ApoEVs served as bone protective factors.^111^ Zhu *et al.* reported that ApoEVs derived from BMSCs enriched in the femur and significantly alleviated bone loss induced by primary (aging) and secondary (ovariectomy) osteoporosis, showing increased bone mass and improved bone microarchitecture. And they further provided with possible mechanisms for BMSC-ApoEVs promoting osteogenesis and inhibiting osteoclast formation, by releasing miR1324, inhibiting expression of the target gene Sorting Nexin 14 (SNX14) and activating the SMAD1/5 pathway.^112^ To improve the bone binding capacity, BMSC-ApoEVs were modified with a bone-targeting peptide, (Asp-Ser-Ser)6 ((DSS)6), onto the surface, and then loaded ubiquitin ligase RING finger protein146 (RNF146), which promoted osteogenesis and alleviated osteoporosis by mediating Axin degradation and consequently activating the Wnt/β-catenin pathway.^113,114^

Furthermore, CDNs carrying intrinsic therapeutic cargos or encapsulating anti-resorptive agents could enhance the therapeutic efficacy in osteoporosis by enabling sustained release and targeted delivery to bone tissue. Study from Cui *et al.* showed that due to their unique endogenous miRNA cargos, EMs generated from endothelial cells facilitated the osteogenic differentiation of BMSCs and alleviated the Mi macrophage dominant microenvironment in osteoporotic bones.^57^ Recent findings revealed that PLT-ApoEVs can rescue bone loss related to osteoporosis *via* Golgi phosphoprotein 2 (GOLPH2)-induced AKT phosphorylation, leading to the therapeutic effects in osteoporosis treatment.^89^ Kang *et al.* demonstrated that MSCs derived EMs equipped with phosphatidylserine (PS) lipid, a native lipid membrane of apoptotic cells, achieved excellent affinity for osteoclast precursors and exerted anti-resorptive effects after encapsulating AMG487-chemical antagonist of CXCR3, an essential axis for osteoclast regulation.^115^ Similarly, MSC-EMs loaded with miR-26a specifically targeted glycogen synthase kinase-3β (GSK-3β) to induce β-catenin accumulation and activated Wnt signaling pathway, showing OVX induced osteoporosis prevention.^60^ In addition, a recent study introduced engineered exosomes (BT-Exo-si*Shn3*) derived from MSCs, that leveraged both the intrinsic anti-osteoporosis functions and exogenous siRNA of the *Shn3* gene, downregulating RANKL expression to inhibit osteoclastogenesis. Notably, BT-Exo-si*Shn3* increased slit guidance ligand 3 (SLIT3) production and particularly restored the formation of type H vessels, which effectively coupled osteogenesis with angiogenesis and were characteristically reduced in osteoporosis.^116^

### 4.3 Periodontitis treatment

The periodontitis microenvironment poses unique challenges for clinical treatments, characterized by reactive oxygen species (ROS) accumulation, persistent inflammatory cytokine expressions, and extensive pathogenic bacteria colonization.^117^ The destructive host microenvironment generates tissue breakdown products and reinforces the inflammation cascade, forcing a positive feedback loop, which further obstruct periodontitis treatment. Therefore, CDNs loaded with therapeutic agents have a demonstrated ability to target periodontal tissue or inflammatory sites, thus achieving a synergistic treatment for periodontitis that is a novel system for delivering nanodrugs.

One the one hand, CDNs exhibited unique bacterial and inflammatory targeting abilities, which efficiently reshaped the hostile periodontitis microenvironment for subsequent regeneration. Conventional antimicrobial agents cannot effectively penetrate the full depth of the biofilms and retain the periodontal tissue interface.^118^ CDNs offer several properties to overcome the periodontal biofilm barriers, such as natural surface ligands enabling tissue-specific retention. Lin *et al.* developed toll-like receptor enriched macrophage membranes anchored with LL-37, the active part of a promising anti-microbial peptide, *via* genetic engineering, which facilitated the specific bacteria elimination and homeostasis restoration. Subsequently, l-amino acid oxidase (LAAO) with hollowed manganese dioxide (hMnO_2_) was incorporated into LL-37 modified membrane nanovesicles to accelerate oxygen production, reduce oxidative stress response and remodulate the macrophage polarization, therefore boosting the alveolar bone regeneration.^119^ Additionally, the biological membrane of CDNs can fuse with bacterial and host cell membranes, facilitating cargo delivery by membrane fusion. Furthermore, CDNs can be functionalized with charge-switching polymers to allow electrostatic interaction with negatively charged membranes of bacteria and endogenous cells. Song *et al.* constructed PLGA/β-tricalcium phosphate (β-TCP) microspheres, osteogenic particles, and coated with poly-lysine (PLL), which was positively charged and could bond to negatively charged macrophage membranes (MM) to fabricate MM@PPT. Particularly, MM, activated by LPS and interferon-γ (IFN-γ), presented maximal number of inflammatory factor receptors and can effectively neutralize inflammatory factors. The results showed that MM@PPT modulated macrophage phenotype, promoted the osteogenesis and mediated the crosstalk between macrophages and BMSCs, resulting in alveolar bone regeneration in periodontitis.^120^ Similarly, macrophages pretreated with *Porphyromonas gingivalis* (P.g.) LPS could derive TLR2/1 enriched membrane to target and directly kill P.g. and disrupt specific binding of P.g. to immune cells.^121^ In a previous study, Wang *et al.* isolated CXCR4-enriched the MC-3T3 cell membrane using genetic transfection and encapsulated curcumin, an effective natural anti-inflammatory compound. This significantly improved homing capacity and ameliorated apical periodontitis.^122^ Pan *et al.* loaded minocycline, an efficient broad-spectrum antibiotic, into polydopamine (PDA) NPs, and then coated NPs with gingival fibroblast membranes, because of the gingival fibroblast's capacity for simple and scalable expansion and the inflammation targeting capacity of their membranes. Afterwards, arginine-glycineaspartic acid cyclic peptide (cRGD) was used to prolong the retention of NPs in the periodontal pocket, as it can bind to the upregulated integrin under inflammatory conditions. And the results showed that these innovative NPs rescued impaired human periodontal ligament stem cells through antioxidant, anti-inflammatory, antibacterial bioactivity, and pro-osteogenic effects *in vitro*, promoted periodontal tissue regeneration and remodeled periodontal homeostasis *in vivo*.^123^ Interestingly, Cao *et al.* isolated bacterial membrane from *S. gordonii*, initial colonizing bacterium supporting P.g. to form symbiotic biofilms, and coated H_2_O_2_ self-supplied nanocomposites, which enhanced internalization by *S. gordonii* and accelerated clearance of biofilms with active hydroxyl radicals derived from encapsulating nanocomposites.^124^

On the other hand, intrinsic therapeutic cargos also contributed to the efficacy for periodontitis treatment. Jing *et al.* conducted a review of the role of MSC-EVs in periodontal regeneration, elucidating their interplay between MSCs and various immune cells (macrophages, dendritic cells, and T cells) and highlighting their capacities to promote osteogenesis, angiogenesis, and cementogenesis.^125^ Ma *et al.* reported that exosomes derived from DFSCs promoted osteogenesis *via* p38 MAPK signaling *via* phosphorylation, and new periodontal ligament-like structures and new bone formation were observed after transplantation in the periodontitis area in rats.^126^ Similarly, pretreatment enhanced the CDNs therapeutic capacities. Exosomes derived from LPS-preconditioned DFSCs inhibited intracellular ROS, reduced the RANKL/OPG ration *via* ROS/JNK signaling inhibition, and promoted M2 macrophage polarization *via* ROS/ERK signaling, improving the therapeutic efficacy for periodontitis in canines.^127^ Zhou *et al.* developed a metal-polyphenol network, specifically tannic acid (TA)-Fe^3+^, as a delivery vehicle for MSC derived EMs, to protect EMs from environmental stimuli at periodontitis sites and reshape the inflammatory microenvironment through their antibacterial, antioxidative, and anti-inflammatory effects, leading to significantly improved periodontal regeneration.^128^ Yu *et al.* demonstrated that myeloid-derived suppressive cells membrane vesicles (MDSCs-MV) exhibited anti-inflammatory properties by inhibiting T cells through the CD73/CD39/adenosine signal pathway. And when applied with PDLSCs in 3D-printed bioink, they significantly enhanced the periodontal bone regeneration with excellent anti-inflammatory efficacy.^129^ Furthermore, Fan *et al.* innovatively isolated exosomes from Schwann cells (SC-Exo), the most principal glial cells myelinating axons in the peripheral nervous system, and observed that these exosomes induced axonal growth of dorsal root ganglia explants, remodulating M2 macrophage polarization and facilitating nerve regeneration in hyperglycemic periodontal microenvironment. Subsequently, grape seed polyphenols (GSPs) were used to coat the surface of SC-Exo through polyphenolic polymerization, which functioned GSP-coated SC-Exo with antibacterial and antioxidative properties, and reversed diabetic periodontal destructive.^50^ Additionally, a preliminary phase 1 clinical trial (ClinicalTrials.gov identifier: NCT04998058) used exosomes from ADSCs isolated autogenously from the patient and injected them locally into the periodontal pockets to evaluate their therapeutic effects in patients with advanced periodontitis (stage III or IV). However, the detailed progression of this clinical trial has not been updated yet.

### 4.4 Dentin–pulp complex regeneration

Dental pulp is a highly vascularized tissue in the root canal surrounded by dentin walls, connecting to surrounding tissue solely through the apical foramen.^130^ As dentin and pulp originate from the dental papilla and have irrelated functions, the dentin–pulp complex could be recognized as a structural integrity, maintaining the sensory and nutritional functions.^131^ The pulp tissue regeneration is mainly due to the cell-homing effect, where dental stem cells are recruited into the empty root canal and form dentin–pulp like tissue.^132^ It has been shown that the key factors for dental pulp regeneration is vascularization driven by hypoxia, where dental pulp cells release proangiogenic factors, with VEGF being the most potent one.^133^

Recent studies have revealed the roles of CDNs in dentin–pulp complex regeneration *via* different mechanisms, but the research mainly focused on exosomes, characterizing their abilities to orchestrate the signaling pathways for angiogenesis and odontogenesis. DPSCs derived exosomes promoted HUVEC proliferation, proangiogenic factor expression, and tube formation through p38 mitogen-activated protein kinase (MAPK) signaling inhibition.^134^ Ganesh *et al.* isolated exosomes derived from DPSCs cultured under angiogenic differentiation condition, which significantly enhanced angiogenic gene expressions, including vascular endothelial growth factor A (VEGFA), Fms-related tyrosine kinase 1 (FLT1), and platelet and endothelial cell adhesion molecule 1 (PECAM1).^135^ Li *et al.* obtained ApoEVs derived from human deciduous pulp stem cells, which promoted endothelial cell autophagy by transferring mitochondrial Tu translation elongation factor (TUFM), activated the angiogenic potentials of endothelial cells and facilitated the formation of dental pulp-like tissue rich in blood vessels in a beagle model.^136^ Interestingly, Chen *et al.* developed a complex model implanted in nude mice to mimic the clinical situation after pulp extraction, where a scaffold loaded with exosomes filled in the treated dental matrix of swine, and the apical dental papilla (SCAPs) were placed only at the tip of each root.^137^ The results showed that exosomes derived from dental pulp tissue promoted the migration, proliferation and differentiation of SCAPs, and recruited SCAPs to regenerate dental pulp-like tissue containing new vessels, odontoblasts, collagen and predentin-like tissue *in vivo*.^137^ Similarly, Zhuang *et al.* derived exosomes from SCAPs (SCAP-Exo) and introduced them into a root fragment containing BMSCs and transplanted into immunodeficient mice, where dental pulp-like tissue presented and newly formed dentine deposited onto the existing dentine in the root canal.^138^ Shi *et al.* demonstrated that MSC exosomes increased DPCs migration, proliferation and odontogenic differentiation by exosomal CD73-mediated adenosine receptor activation of AKT and ERK signaling, resulting in the formation of dentin-like tissue and bridge-like structures in a rat pulp defect model.^139^ Particularly, Sun *et al.* engineered dental pulp cells to display high expression of TLR4 with LPS stimulation, and derived cell membranes to coat PLGA nanoparticles as an early-stage treatment of pulpitis, where the upper molar was occlusally exposed and applied with *Escherichia coli* lipopolysaccharide (*E. coli* LPS) to induce early pulpitis in rats.^140^ The results showed that these nanoparticles could neutralize invasive *E. coli* LPS *via* TLR4 on the DPCs membrane, alleviating expression of multiple inflammatory cytokines and attenuating inflammatory conditions.^140^

Several studies immobilized nanovesicles into functional scaffolds with improved mechanical properties for dental pulp regeneration. Tsai *et al.* developed a hydroxyapatite/gelatin (HAp/Gel) scaffold loaded with DPSC-Exo, which was applied to the exposed pulp and covered with glass ionomer cement for coronal sealing in rat maxillary molars, showing enhanced mineralized tissue formation and dentin bridge development.^141^ Additionally, exosomes isolated from hDPSCs were embedded into a hydroxypropyl chitin (HPCH)/chitin whisker (CW) thermosensitive hydrogel, which could be easily injected into irregular endodontic spaces and gelated *in situ*.^142^ Results showed that this exosome loaded hydrogel could promote odontogenesis and angiogenesis *in vitro* and formed new dental pulp-like tissue in an implanted tooth root model.^142^

Furthermore, Jafari *et al.* reported a clinical case that hUCMSCs derived exosomes were applied to the root canal of a mandibular second premolar after pulpectomy in a 40-year-old patient. Clinical follow-ups over 24 weeks showed no signs of infection, swelling or tenderness, and radiographic assessment indicated active healing.^143^ And an ongoing clinical trial (ClinicalTrials.gov identifier: NCT06764004) aimed to utilize exosomes derived from UCMSCs for regenerative endodontic treatment of necrotic open apex molar teeth and compared their therapeutic efficacy with UCMSCs.

## 5. Challenges and future directions

In recent decades, research on CDNs has remarkably expanded our understanding of cell-free therapeutic strategies for oral and craniofacial regeneration, offering a promising alternative to traditional stem cell-based therapies. By inheriting the bioactive components from their parent cells, CDNs possess the unique capabilities that are strictly cell-source-dependent. Therefore, CDNs derived from competent therapeutic cells can restore microenvironmental homeostasis and promote tissue reconstruction at the injured and impaired sites. Importantly, this cell-free approach mitigates the major limitations associated with live cell transplantation, such as tumorigenicity and low cell survival rates. However, despite the increasing number of studies showing experimental success, a critical evaluation of the existing literature reveals several methodological bottlenecks that must be addressed to strengthen the translational evidence base. Besides, the translation of CDNs from promising bench-top findings to reliable bedside solutions continues to be hindered by substantial obstacles. To realize the full clinical potential of this technology, the field needs to address the limitations surrounding standardized production, develop long-term safety profiles, and understand the mechanism of their therapeutic effects. In this section, we will focus on these pivotal challenges and outline the future directions necessary to achieve CDN therapeutics toward regulatory approval and clinical application in craniofacial regeneration.

### 5.1 Critical appraisal of current pre-clinical evidence

For pre-clinical studies cited from high quality journal in this review, fascinating outcomes prove the regenerative potentials in oral and craniofacial tissue, while evidence need to be further evaluated across the following dimensions. First, this field suffer from a heterogeneity in dosing metrics and normalization strategies. Current studies inconsistently report CDN doses based on total protein mass, particle number quantified by NTA, or parent-cell equivalents, which precludes cross-study comparisons regarding therapeutic efficacy and pharmacokinetics. Regarding animal model relevance, the majority of these pre-clinical studies use small rodent models, which often fail to accurately recapitulate the complex immune microenvironment, biomechanical loading, and microbial characteristic of human diseases. Particularly, the ligature-induced periodontitis model differs from the chronically progressive human periodontal disease. Finally, another critical limitation is the lack of appropriate positive controls. While many investigations demonstrate CDNs superiority over negative controls (*e.g.*, PBS, saline), there remains a critical need to compare CDNs against conventional clinical approaches, such as The Food and Drug Administration (FDA)-approved recombinant growth factors or traditional autogenous bone grafts for bone regeneration. It is essential to validate the regenerative benefits of CDNs exceeding those conventional strategies, which is a meaningful translational benchmark for clinical-scale manufacturing.

### 5.2 Reproducibility and scalability

Various techniques have been applied to successfully fabricate CDNs, reliant on specialized expertise and equipment, and may lead to low external validity. Notably, composition and function of CNDs depend on cell source, culture condition and passage number, resulting in inconsistent regenerative outcomes. Establishing standardized manufacturing approaches must be developed to decrease heterogeneity and improve the reproducibility. Production costs of CDNs are associated with cell culture, isolation and characterization, and bioactive agents for modification, which pose potential challenges for scale-up productions. Even though EMs have yields that are 10 times that of exosome production, their cost still hinders clinical grade applications. To overcome these challenges, developing novel fabrication methods is essential for reproducible, scalable and cost effective for scale up productions.

### 5.3 Safety and immunogenicity concerns

Unlike synthetic materials, CDNs are natural components derived from cells with inherent biocompatibility and low toxicity. However, their clinical translation must address systemic risks related to procoagulant activity and immunogenicity. For example, Rognes *et al.* reported that a massive endogenous release of circulating EVs induce a transient hypercoagulable function in individual trauma patients, while EV-associated thrombin generation normalized within 7 to 12 hours after injury.^144^ Given this intrinsic procoagulant capacity of natural EVs, the administration of concentrated therapeutic CDNs must be carefully monitored for similar thrombotic risks. Besides, EVs inherit major histocompatibility complex (MHC) antigens from their parent cells, participating in antigen presentation with important roles in immune regulation.^145^ Thus, repeated administration of allogeneic CDNs may carry a risk of adverse immunogenic reactions, provoking an immune response, and disrupting microenvironment homeostasis. Besides, prolonged retention in the body may lead to potential accumulation effects and chronic inflammatory reactions. Rigorous evaluations are necessary to comprehensively assess the effects on the immune system, long-term biodistribution, and potential risks with undesired oncogenic nuclei acid. Additionally, it is crucial to eliminate the denaturation of membrane biomarkers from CDNs isolation and engineering approaches to prevent potential immune responses by endogenous antigens.

### 5.4 Lack of mechanism elucidation

The restoration of the craniofacial tissue is hindered by a complex interplay of oxidative stress, persistent inflammation, and dysregulated immune responses. While CDNs exhibit multiple biofunctions, including the restoration of homeostasis, immunomodulation, and potent antioxidant activity, the mechanisms of their therapeutic effects on the local microenvironment remain unclear. The ‘black box’ nature of CDN-mediated regeneration stems from the intricate synergy among encapsulated miRNAs, proteins, and lipids, making it difficult to isolate specific signaling axes. Furthermore, the prevalent use of xenogeneic or allogeneic cell sources in current research introduces significant interfering variables, necessitating a more rigorous elucidation of signaling pathways before broad clinical translation.

### 5.5 Future directions

While the CDNs discussed herein share a foundation of biological membranes and bioactive cargo, their distinct origins result in divergent pathways for clinical manufacturing and regulatory approval. From a clinical-scale production perspective, naturally secreted exosomes and ApoEVs face critical bottlenecks in upstream scalability and downstream yield. Conversely, engineering platforms, EMs and mNVs, offer highly scalable manufacturing yet present significant Chemistry, Manufacturing, and Controls (CMC) challenges regarding internal cargo heterogeneity and the preservation of native protein orientation. Furthermore, navigating these diverse platforms requires different regulatory frameworks. Purely biological vesicles, exosomes and ApoEVs, are most likely to be regulated as biological medicinal products, necessitating batch-to-batch consistency and purity metrics. In contrast, EMs and engineered mNVs often fall under the regulatory pathways of combination products or nanomedicines. This requires tailored CMC strategies to successfully navigate the path from bench to bedside, demanding compliance with both medical device and biologics regulatory frameworks. Except for the four clinical scenarios we mainly delve into, there are several clinical trials evaluating the therapeutic effects of CDNs on other oral and craniofacial related situations, including relieving craniofacial neuralgia (ClinicalTrials.gov identifier: NCT04202783, NCT05152368), preventing and treating oral mucositis associated with chemoradiation treatment for head and neck cancer (ClinicalTrials.gov identifier: NCT01668849, NCT07312656), showing potentially broad range of clinical applications in oral and craniofacial tissue regeneration. However, several critical milestones must be achieved before CDNs’ integration into routine clinical practice.

Foremost among these is the standardization of isolation and manufacturing protocols, which is essential for scalable production. Currently, the diverse array of methodologies used to generate CDNs results in variability to a certain extent regarding their composition and biological functions across different existing studies. Therefore, thoroughly characterizing and addressing this inherent heterogeneity is critical to strengthen the reproducibility and validity of pre-clinical outcomes. Future efforts should transition from small-scale laboratory techniques to Current Good Manufacturing Practice (CGMP) – compliant processes to ensure batch-to-batch reproducibility, quality control, and scalability. Establishing a global consensus on CDN isolation for craniofacial tissue regeneration is imperative, as variables such as cell source, culture conditions, and fabrication methods significantly influence downstream applications and therapeutic outcomes.

Beyond manufacturing considerations, future research must prioritize comprehensive evaluations of long-term efficacy and safety. Although existing studies demonstrate encouraging short-term regenerative outcomes, longitudinal investigations are required to fully characterize tissue remodeling, biodistribution, and systemic metabolic clearance of nanovesicles over extended time frames. In addition, there is a critical need to establish disease models that more accurately replicate clinical scenarios for long-term evaluation. For example, the pathophysiology of craniofacial diseases such as periodontitis and pulpitis is substantially more complex than that represented by currently available animal models, which fail to fully recapitulate human clinical conditions. Consequently, further studies employing advanced and clinically relevant models are necessary to rigorously evaluate therapeutic efficacy and to elucidate the underlying biological mechanisms of CDN-mediated regeneration.

Currently, overall regenerative efficacy remains suboptimal. Advanced bioengineering strategies should therefore be leveraged to enhance CDN performance, including surface modification, improved drug-loading efficiency, and controlled release from biomaterial scaffolds. Specifically, next-generation CDNs must advance toward precise tissue targeting. Functionalization of vesicle surfaces with tissue- or disease-specific ligands could enhance targeted delivery and retention at defect sites. For successful clinical translation, minimizing potential immunogenicity is also critical. A strategic shift toward autologous sourcing—using a patient's own progenitor cells to generate personalized CDNs—may help circumvent host immune responses and improve safety profiles. Furthermore, navigating complex regulatory frameworks is a prerequisite for the successful clinical translation of CDNs. Although these nanovesicles present significantly lower immunogenic and ethical risks compared to traditional live-cell therapies, strict regulations remain necessary to mitigate potential hazards, such as infectious disease transmission, and to ensure standardized safety profiles.

Fortunately, the development of artificial intelligence (AI) has offered numerous non-destructive solutions for advanced CDNs. In terms of standardization and quality control, Machine Learning algorithms can optimize upstream technique parameters by predicting the ideal cell culture and CDN fabrication conditions to maximize yield and reduce batch-to-batch variability. Manufacturers can identify critical quality attributes (CQAs) in real-time, including CDN size, concentration, purity, content uniformity, and cargo characteristics, by training Deep Learning algorithms on high-resolution imaging and biological data. To elucidate this ‘black box’ of CDNs, machine learning models can be utilized to analyze the complex interactions among the thousands of bioactive cargos encapsulated within CDNs, mapping these molecular fingerprints to specific regenerative outcomes to identify the key ‘driver’ signaling networks responsible for tissue regeneration. Furthermore, the convergence of AI and bioengineering enables the ‘digital twin’ simulations in oral and craniofacial regeneration. Before treatment, virtual models of patient-specific defects and CDNs can be simulated to predict the optimal type and modification of CDNs and biomaterial carrier for patients individually.

Collectively, these innovations, particularly when combined with responsive and intelligently designed scaffolds, have the potential to redefine the clinical management of complex craniofacial regenerative challenges.

## 6. Conclusion

In conclusion, CDNs represent a transformative strategy in craniofacial regeneration, offering outstanding advantages in precise tissue targeting and the accurate delivery of bioactive agents. While robust preclinical evidence demonstrates their therapeutic efficacy, bridging the gap to clinical translation requires efforts toward standardizing manufacturing, long-term safety and efficacy evaluation in advanced disease models. Ultimately, the integration of CDN-based therapies into clinical practice holds the potential to improve the care for complicated oral and craniofacial diseases to a higher level and significantly enhance patient outcomes and quality of life.

## Author contributions

L. Zhong: investigation, writing – original draft. J. S. Marschall: visualization, writing – review & editing. K. Shin: visualization, writing – review & editing. H. Sun: supervision, resources, funding acquisition, writing – review & editing.

## Conflicts of interest

There are no conflicts to declare.

## Abbreviations

ADSC: Adipose-derived stem cell
ApoEV: Apoptotic extracellular vesicles
ATP: Adenosine triphosphate
BMDM: Bone marrow derived macrophage
BMEC: Bone marrow endothelial cell
BMP-2: Bone morphogenetic protein-2
BMSC: Bone marrow derived mesenchymal stem cell
CBCT: Cone beam computed tomography
CDN: Cell-derived nanovesicles
DFSC: Dental follicle stem cell
DPSC: Dental pulp stem cell
EM: Exosome mimetic
ERK: Extracellular regulated kinase
Exo: Exosome
GelMA: Gelatin methacryloyl
GF: Gingival fibroblast
HA: Hydroxyapatite
HUVEC: Human umbilical vein endothelial cells
IFN: Interferon
IL: Interleukin
LPS: Lipopolysaccharide
MAPK: Mitogen-activated protein kinase
miRNA: MicroRNA
mNV: Membrane nanovesicles
MSC: Mesenchymal stem/stromal cell
OPG: Osteoprotegerin
OVX: Ovariectomy
PDLSC: Periodontal ligament stem cell
P.g.: *Porphyromonas gingivalis*
PLGA: Poly(lactic-*co*-glycolic acid)
RANK: Receptor activator of nuclear factor-κB
RBC: Red blood cells
ROS: Reactive oxygen species
SC: Schwann cell
TLR: Toll-like receptors
TNF-α: Tumor necrosis factor alpha
UCMSC: Umbilical cord mesenchymal stem cell
VEGF: Vascular endothelial growth factor

## Data Availability

Data sharing is not applicable to this article as no new data were created or analyzed in this study.
